# Supplementary figures and images for: Electrostatic and bending energies predict staggering and splaying in nonmuscle myosin II minifilaments
Source: PLoS Comput Biol. 2020 Jul 6;16(7):e1007801. doi: 10.1371/journal.pcbi.1007801 (PMC7365473; doi:10.1371/journal.pcbi.1007801)

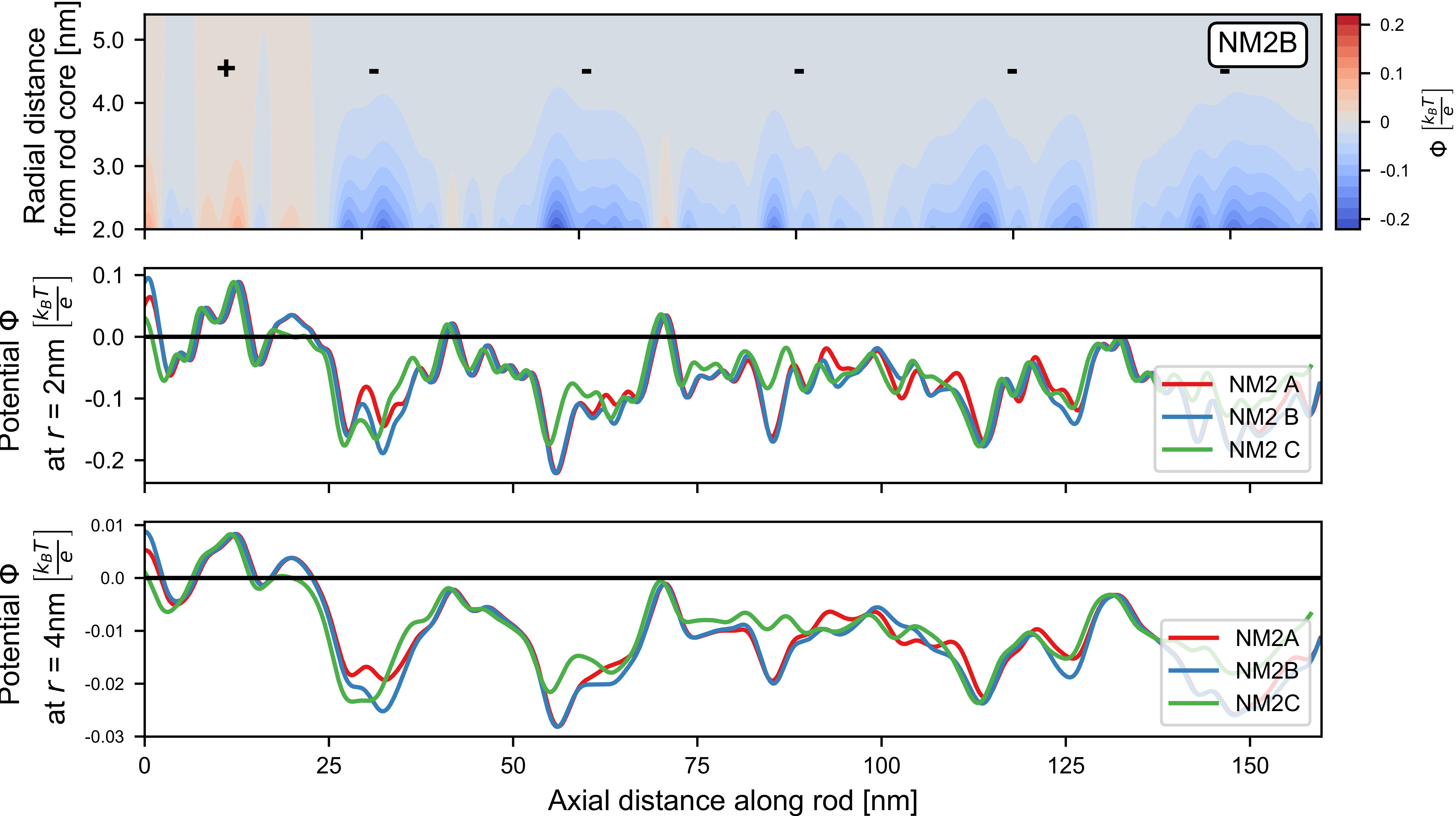

Supplement: S1 Fig — The NMII-rod was treated as a linear chain of charges and the electrostatic potential calculated using the Debye-Hückel theory (see Materials and methods). The subfigures show the electrostatic potential along the axis for variable distance radial distance r = (top), r = 2 nm (middle) and r = 4 nm (bottom). The positively charged ACD as well as the five regions of increased net negativity are clearly visible for all three isoforms. (TIF) [file pcbi.1007801.s001.tif]

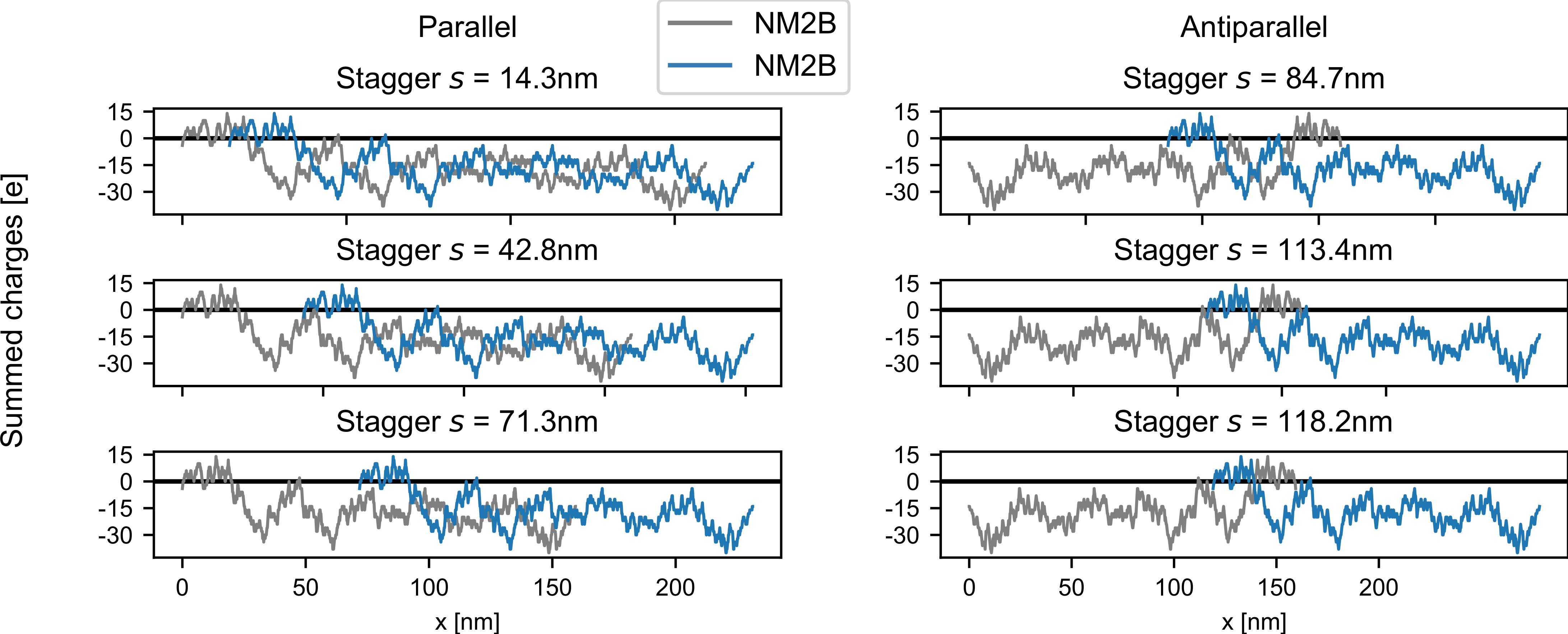

Supplement: S2 Fig — The charge distribution was calculated using a sliding window technique; the rod was treated as a linear chain of charges and the charges summed over a window of 98 charges. It is clearly visible how the experimentally observes staggers correspond to interactions between the positively charged ACD and the regions of increased net negativity. (TIF) [file pcbi.1007801.s002.tif]

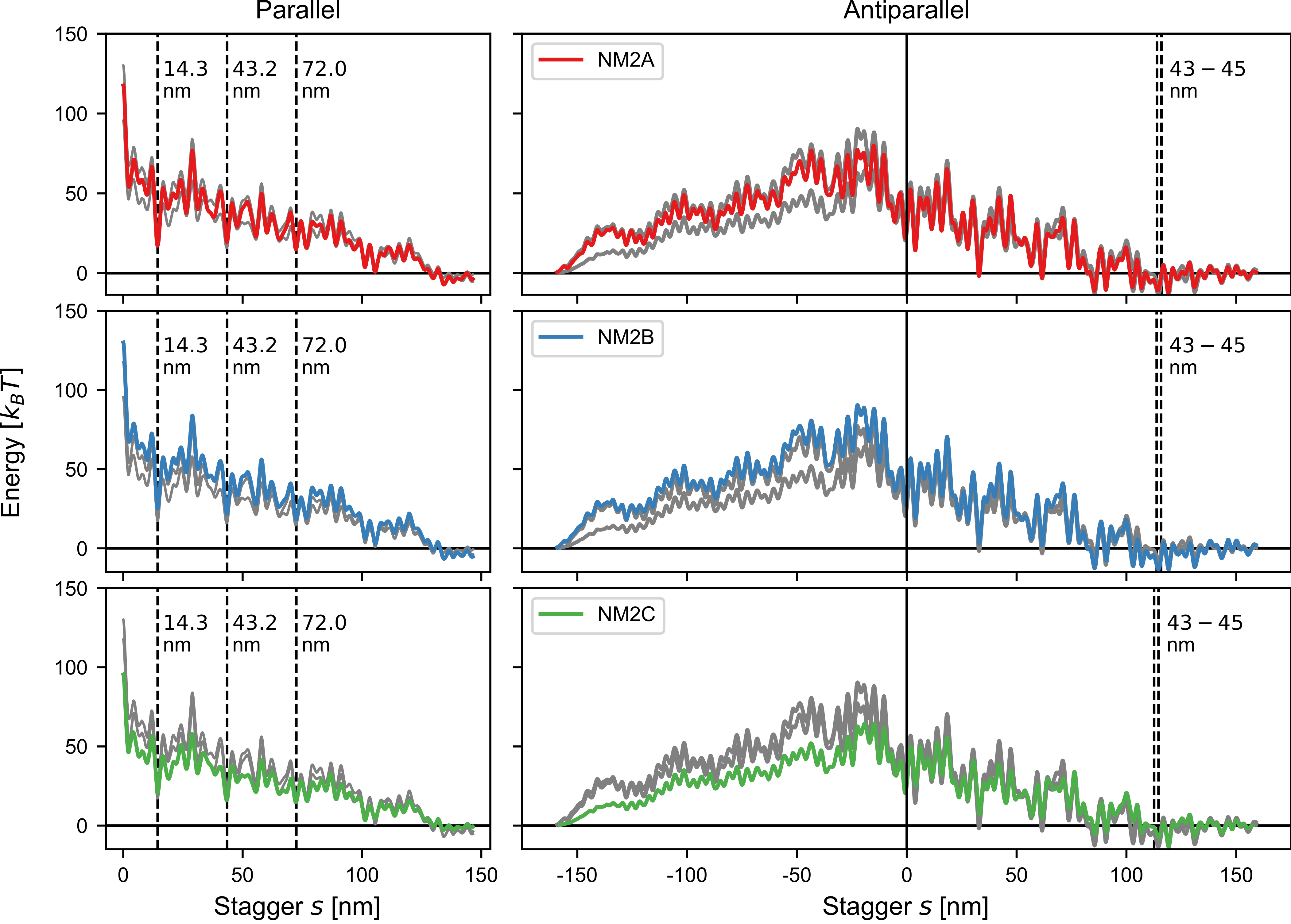

Supplement: S3 Fig — While Fig 1D shows the results for NM2B, here we show them for all three isoforms. The three prominent parallel staggers and the one prominent antiparallel stagger are the same for all three isoforms. (TIF) [file pcbi.1007801.s003.tif]

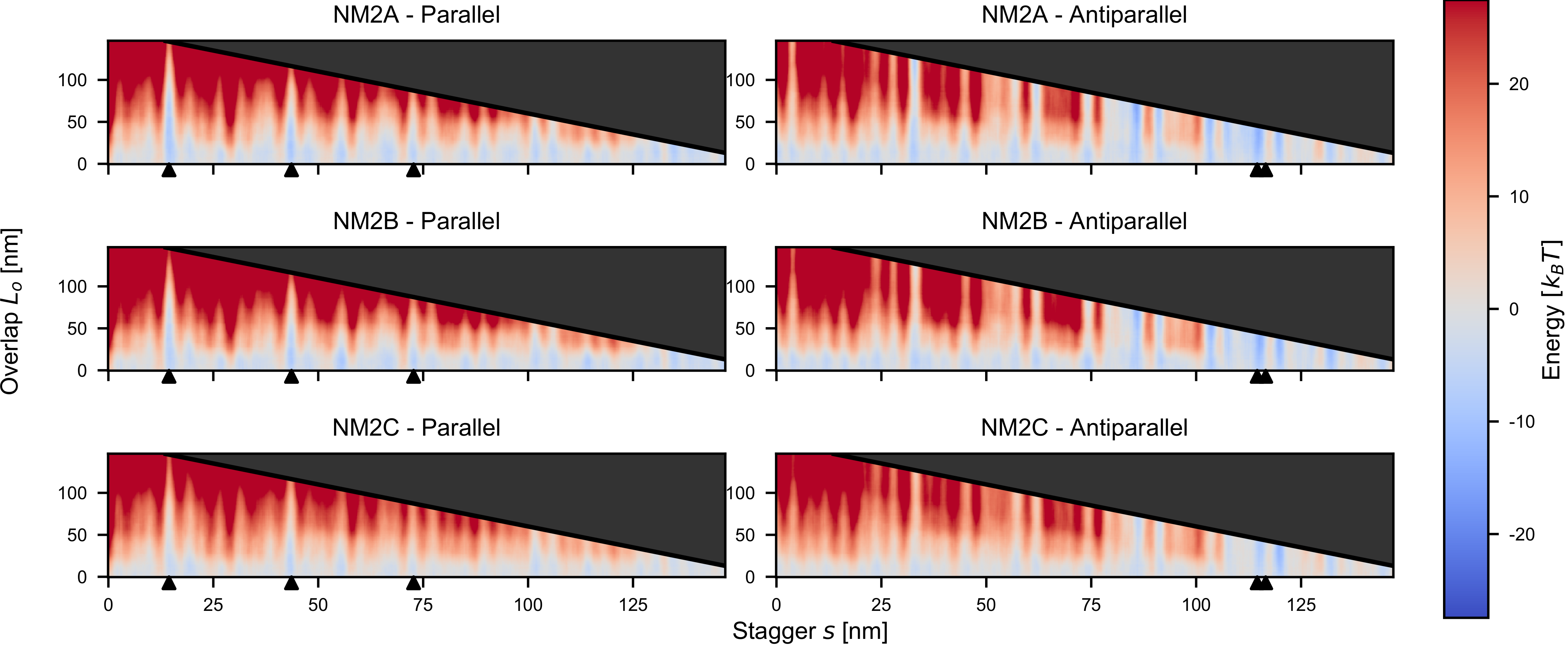

Supplement: S4 Fig — While Fig 2B shows the results for NM2B, here we show them for all three isoforms. Again the same known staggers emerge for all three isoforms. (TIF) [file pcbi.1007801.s004.tif]

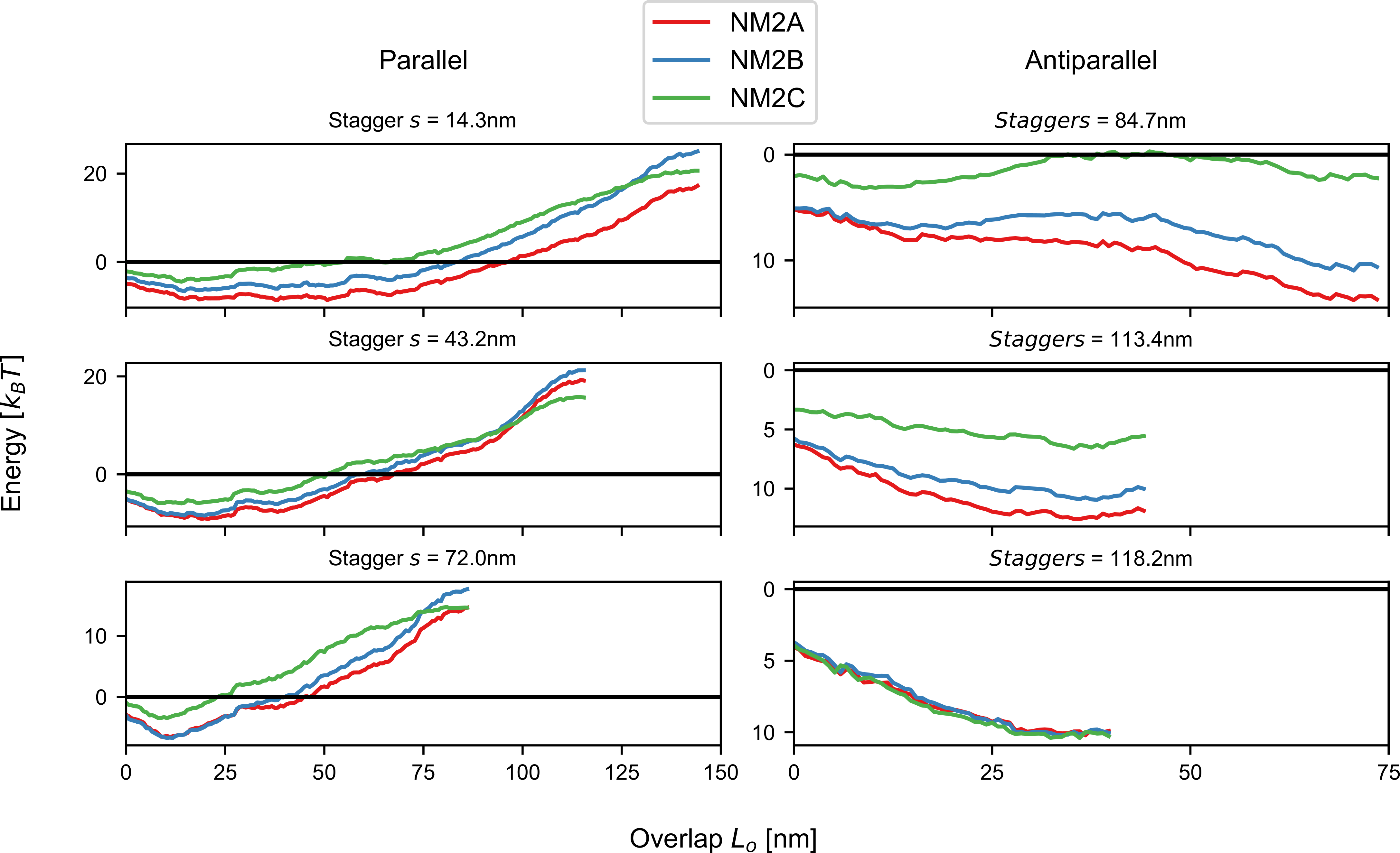

Supplement: S5 Fig — While Fig 3 shows the results for NM2B, here we show them for all three isoforms. These plots correspond to vertical cross-sections of S4 Fig. Again all isoforms show similar behavior. (TIF) [file pcbi.1007801.s005.tif]

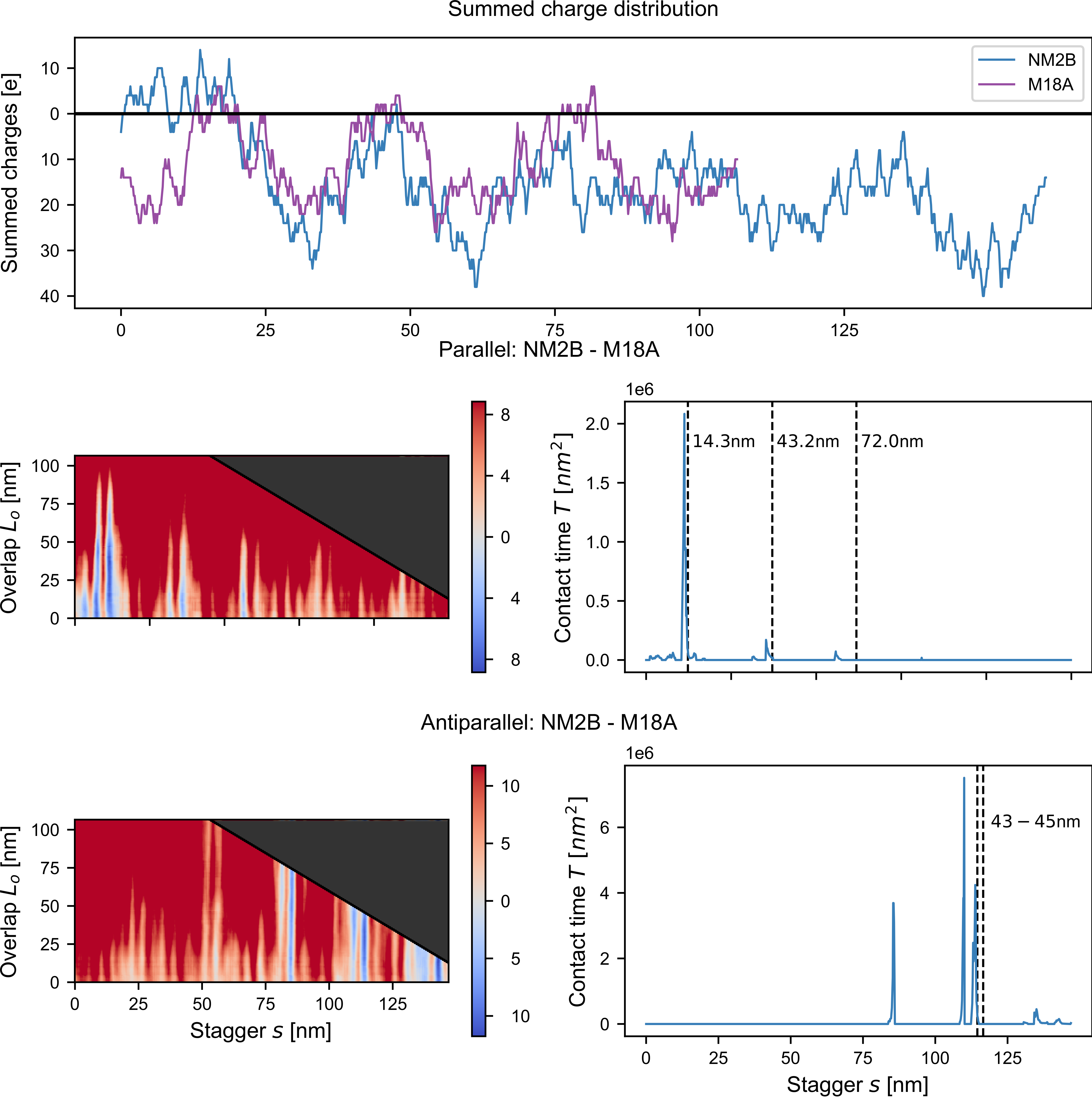

Supplement: S6 Fig — At the top we show a comparison of the charges along the respective rods using the sliding window technique (compare S1 Fig. The middle shows the total energy for parallel and antiparallel interactions. Here, the NMIIB remains straight while the M18A can bend away. The bottom plot shows the contact times between NMIIB and M18A with respect to the staggers. The most stable configurations are close to the experimentally observed values. There are no favorable large parallel or small antiparallel staggers, which is line with the experimental observation that M18A localizes at the middle of the minifilaments. (TIF) [file pcbi.1007801.s006.tif]
