# Supplementary material for: Electrostatic and bending energies predict staggering and splaying in nonmuscle myosin II minifilaments
Source: PLoS Comput Biol. 2020 Jul 6;16(7):e1007801. doi: 10.1371/journal.pcbi.1007801 (PMC7365473; doi:10.1371/journal.pcbi.1007801)
Supplement: Attachment — Submitted filename: ResponseToReviewers.pdf [file pcbi.1007801.s007.pdf]

We thank the guest editor, the deputy editor and the two reviewers for their very fair and positive evaluation of our manuscript. We are delighted to learn that publication is likely and below answer the comments and questions by the guest editor and the two reviewers. Title, abstract and figures are unchanged, but we have made several changes to the main text. As suggested, we now upload two versions of the revised manuscript, one in which our changes are marked in red and a clean version. We hope that our manuscript can now be accepted for publication in its revised form.

Response to comments by guest editor

The two reviewers of the manuscript as well as myself found your work important and novel. When you resubmit your manuscript, please consider their minor suggestions as well as the following questions I had:

1) Refs 16, 19, 24, 26 are mentioned as earlier studies where electrostatic energy calculations have been performed. Can you discuss any differences or similarities in terms of magnitude and overall trend (between prior and current energy calculations)? Would this comparison justify the level of coarseness used in this work?

While the main novelty of our work is the incorporation of the rod bending energy and the focus on the dynamic nature of the assembly process, we completely agree that it is also important to describe in detail how our electrostatic calculations differ from earlier work.

From the references mentioned by the guest editor, Ref 16 is standing out because it is the most sophisticated treatment (here we use the reference numbering from the initial submission). Refs 19, 24, 26 used relatively simple models which represent the NM2 rods as linear charge distributions and calculate electrostatics simply by counting the number of like and opposing charges which were opposite each other. Although we also use the representation of the linear charge distribution, our calculation of electrostatic energy is more detailed, because we use the Debye-Hückel equation. Refs 19, 24, 26 found the experimentally observed staggers as local energy minima. However, due to the simple nature of the electrostatic calculations, the results were very noisy and cannot be assigned an absolute value.

In marked contrast to these papers, Ref 16 used a 3D molecular model and a full Poisson-Boltzmann solver for the electrostatic interactions. It should be noted that, as the molecular structure of NM2 is not known, the authors had to use the known structure of a similarly shaped coiled-coil. For this reason, they were also not able to include the three so-called skip residues into their sequences (see below), thereby increasing the regularity of the model structure compared to the real protein. Nevertheless, it is clear that their model is much more detailed than ours. Very importantly, however, a comparison of the shapes of the electrostatic energy landscapes for the straight rods show that in this regard, our calculations match the ones from Ref 16 very closely (compare our Figure 1D and Figure S2A from Ref 16). Not only do we find the same local energy minima at the experimentally observed staggers, but also the overall shape of the energy landscapes are very similar with the main difference being that our model predicts slightly more favorable interactions for large staggers. Ref 16 also found the effect of axial rotations to be negligible, validating our approach of using a linear charge distribution.

Apart from the shapes of the energy landscapes and the predicted staggers, it is also instructive to compare the predicted interaction energies. The absolute energy levels for human NM2 in Ref 16 are given in their Figure S2A: maximal parallel repulsion 300 kcal/mole = 505.9 k_BT, maximal antiparallel repulsion 250 kcal/mole = 422 k_BT and maximal antiparallel attraction 20 kcal/mole = 33.7 k_BT (using k_BT = 0.593 kcal/mole). In contrast, we find overall lower values with maximal parallel repulsion at 120 k_BT, maximal antiparallel repulsion at 100 k_BT and maximal antiparallel attraction of 10 k_BT (compare Figure 1D). So we seem to agree on the relative importance, but the absolute numbers are off by a factor around 4. In fact we think that our lower numbers make sense in light of the fast exchange dynamics measured with FRAP.

There are several possible reasons for this discrepancy. Most importantly, Ref 16 does not specify which ionic strength was used. A different value of the ionic strength could explain the differences in magnitude of the electrostatic energies. Secondly, Ref 16 removed the skip residues, thereby increasing the structural regularity and most likely increasing interaction energies.

As we are not actually interested in the absolute values of the electrostatic energies, but rather in their relative values with respect to the staggers and how they translate to the contact times between two rods, which also makes only sense in a relative manner due to the unknown effective diffusion constant, we believe that the good agreement in terms of the relative binding energies strengthens our conclusions and that the disagreement in the absolute numbers is not a major concern.

In summary, our treatment is more detailed than the ones of Refs 19, 24, 26 due to the use of Debye-Hückel theory, and less detailed than the one of Ref 16 because we do not use a molecular model. Nevertheless our results seem to be quite close to the ones of Ref 16 in regard to the relative importance of the different configurations and we take this is justification for our choice for the level of coarse-graining.

We finally note that the sliding window over 98 residues was only used to demonstrate the global pattern in the charge distribution. No sliding window was used for the calculation of the electrostatic energy, so no further coarse-graining was involved here.

In response to this comment, we now have included a new and very detailed discussion of this subject in the discussion section (second and third paragraphs, page 11, lines 336-358).

2) In line 98 it is stated that "For small ionic strengths, the Poisson-Boltzmann equation can be linearized…" It's not clear if this means that the linearization approximation is valid considering the stated charge density of the myosin tail. Can you comment on this approximation? Also, is the system far from regimes where counterions may cause attraction between rods of same charge?

The potential relevance of counterion condensation is a very valid point as it is for example involved in DNA-compactification. In this case, not even non-linear Poisson-Boltzmann theory would be valid. This would be the case for a coupling constant Ξ larger than 1, where we define Ξ as the ratio of the Bjerrum length and the Gouy-Chapman length (for an overview over the different electrostatic theories, we refer to

the excellent review on “Electrostatic interactions in strongly coupled soft matter” by the group of Roland Netz in Physica A 2005). At room temperature, the Bjerrum length is roughly 0.7 nm in water. For the Gouy-Chapman length we have to estimate the charge density on the surface of the rod. This is not straightforward as the rod is very irregularly charged. In the simplest way we can treat the rod as a cylinder with radius 1 nm, assume a linear spacing of 0.1456 nm between neighboring residues and use the average charge along the rod of -0.085e per residue (as calculated in our model for NM2A) to arrive at a surface charge density of 0.09 e/nm^2. This results in a Gouy-Chapman length of ~11 nm and thus a coupling constant of $\Xi \sim 0.06$. Therefore counterion condensation can be neglected and we can use the mean-field Poisson-Boltzmann theory.

For the linearization of the Poisson-Boltzmann theory the electrostatic potential has to satisfy $e\Phi \ll k_B T$. In our model we do not consider the self-energy of a given rod and thus do not calculate the electrostatic energy between residues of the same rod. For residues between two rods, due to the small Debye-Hücke length of ~1.3 nm and the radius of the rods of 1 nm, we have values of $e\Phi$ far below $k_B T$, so linearization is also justified.

Due to the irregular distributions of charges along the rod, there could potentially be regions where strong coupling and/or breakdown of Debye-Hückel can occur. However, here we refer again to the similarities between our results and the ones obtained by the detailed model in Ref 16 and use these similarities to justify the simplifications made.

We finally note that in the cellular context, ionic conditions and also pH might vary locally. This opens an interesting perspective of myosin II minifilament regulation that could be addressed in future work.

In order to address this important comment, we now have included our estimates on weak coupling and linearization in the materials and methods section (page 3 and 4, lines 99 - 114). We also now cite the review by the Netz group for details on strong coupling, Poisson-Boltzmann and Debye-Hückel theories.

3) Would accounting for the entropic fluctuations of the tail modify any of the results?

This question has different aspects to it. We first note that the entropic fluctuations of the tail are already incorporated as part of the dynamic folding/unfolding of one rod onto another. Here we consider a Fokker-Planck framework in which the dynamic movement contains both the directed movement due to the electrostatic and bending energies (drift term) and the undirected movement due to the entropic fluctuations of the tail (diffusion term). The undirected movement is quantified with an effective diffusion constant D. For the sake of computational simplicity we assume a constant D. Realistically, D would change with the length of the free part of the rod. However, as the electrostatic interactions become extremely unfavorable for large overlaps (with the exception of some antiparallel staggers), the scenario of small free rod lengths is not relevant for our model. Due to the complicated relationship between the entropic fluctuations of the tail and the effective diffusion, we do not assume an actual value for D. However, this should not affect our predictions regarding the relative values for the contact time T.

Another interesting aspect is the effect of entropic fluctuations on the interaction with the environment, in particular for binding to actin filaments. Although a model for the environment is not the subject of our current work, we are happy to comment on this. In order to estimate how much the splaying and its thermal fluctuations lead to spatial exploration of the environment, we again make use of the concept of the persistence length $l_p \approx 130$ nm, which is the length after which thermal fluctuations take over. As before, we restrict bending to circular arcs. The maximum distance of the head to the backbone is achieved if the rods bend along a quarter-circle of arc-length l_p (and therefore radius $R = 2*l_p/\pi \approx 83$nm) and continue straight afterwards. Then we can estimate the transverse distance y of the myosin heads to the rod. For L_o=75, 50, 25 and 10 nm, we get y≈40, 60, 90 and 100 nm, respectively. Thus, for the first stagger at s=14.3 nm with expected overlaps L_o in the range of [25nm,75nm] (see Figure 3), thermal fluctuations alone lead to the heads searching its vicinity at 40-90 nm distance perpendicular to the rods. As the Debye-Hückel length is 1.3 nm, we can neglect the effect of the charges for this argument.

We now have included a new paragraph with these estimates in the discussion (page 12, lines 374-387). There we also comment that future work should include a model for the actin environment.

Response to comments by reviewer #1

The authors described an insightful model that combines continuum electrostatics and elasticity to model the assembly of nonmuscle myosin II minifilament. By minimizing the effective energy of the system with different degrees of staggering and computing the relevant mean first passage time (referred to as "contact time"), the authors correctly predicted the favorable staggerings of parallel and anti-parallel dimers. Moreover, they demonstrated that the relatively simple model can also capture the differences between homodimers and heterodimers observed in recent experiments, supporting the general validity of the model.

Overall, the ms is clearly written with solid connection with experimental observations. Further extension of the model to multiple rods will be able to model more diverse behaviors for helping better interpret experimental observations. I support the publication of the work in PLoS Comp Biol.

We thank the reviewer for this very positive evaluation.

I have only several minor questions/comments:
1. The model assumes a regular coiled-coil structure and therefore canonical charge distribution associated with such structure. In reality, however, there are skip residues that disrupt the regular coiled-coil pattern and modulate both the helical pitch and solvent-exposed residues (see, for example, recent work of I. Rayment, Cui and co-workers). The authors should discuss the potential impact of the skip residues.

We agree that our treatment of the skip residues is an important point that requires more discussion. Despite being only single isolated residues inserted into the heptad repeat (four for skeletal and cardiac myosin II, three for smooth and non-muscle myosin II), skip residues are far more than punctual anomalies but rather extended regions of structural alterations, as shown in the mentioned paper Taylor et al. PNAS 2015 for cardiac myosin II. Skip residues lead to over- or underwinding of the coiled-

coil and are believed to confer increased flexibility and binding capacity to the myosin rod. They also show some correlation with the bends required for the inactive and energy-saving (10S) form of myosin II, which for sarcomeric myosin II leads to the superrelaxed state that saves energy in skeletal and cardiac muscle, and to the transport state for smooth and non-muscle minifilaments, which forms the reservoir for assembly. However, the exact relation between skip residues and these bends is not clear (compare e.g. Burgess et al. J Mol Biol 2007).

It is almost impossible to include the conformational effects of the skip residues in the model of a linear chain of charges. As correctly stated by the reviewer, this inaccuracy has two main implications for our model. Firstly, we assume a regular spacing between neighboring amino acids which is usually granted by the regular coiled-coil structure but disrupted in the vicinity of the skip residues. Thus, our model incorrectly treats the exact charge distribution around the skip residues and can result in a shift of the charge distribution of our model with respect to the actual protein structure.

Secondly, the changed structures around the skip residues disturb the heptad pattern of residues, as a result of which side chains that would be predicted to be solvent exposed are now buried along the interface and vice versa (as also discussed in Taylor et al. PNAS 2015). In our model we calculate the position of residues within the heptad repeat using the software paircoil and neglect charges at positions *a* and *d* as they are buried inside the coiled-coil structure. The software is not able to correctly classify amino acids around the skip residue which likely results in errors in the charge distribution of our model around the skip residues. Another consequence of these conformational changes might be that hydrophobic residues contribute more to staggering than assumed here. However, electrostatics is known to dominate assembly because it is strongly salt-dependent. Moreover, a large role of hydrophobic residues would break the rotational symmetry of the coiled-coil staggering, which is generally believed to be an essential feature of minifilament architecture.

Despite the listed shortcomings regarding the exact treatment of the skip residues, we believe that they do not affect our conclusions. In a coarse-grained model like ours, it is difficult to treat these structural details and the main issues as described here could only be addressed in a molecular model, e.g. starting from molecular dynamics (like in the paper by Taylor et al. PNAS 2015, but for larger system size) and then coarse-graining these results.

We note that the molecular model for staggering by Ricketson et al. PNAS 2010 did not treat the skip residues, because they perturb the regular coiled-coil structure that is assumed in their work. In our work, we actually include the skip residues, because otherwise we would obtain an unrealistic high degree of order in the coiled-coil interactions. Future work has to strike a balance between these different approaches.

In response to this very important point, we now mention and discuss the skip residues at several positions in the text. On page 3 in the materials and methods section (lines 89-96), we explain in more detail how we treat them and why. On page 3 and 4, we mention them briefly in the context of electrostatics. In the discussion we have added a completely new paragraph (last paragraph, on page 14, lines 459-476)

discussing potential effects of skip residues. Here we also cite the Burgess paper and state that future work should address the exact role of the skip residues.

2. Are there predictions that the authors can make for possible experimental tests?

This question is again well taken yet challenging. Myosin II minifilaments are positioned exactly in the grey zone between being too large for detailed electron microscopy and being too small to be seen well with optical microscopy. Nevertheless both methods are currently fast improving and in the future might be able to provide direct visualization of the processes predicted here. If sample preparation was sufficiently structure-preserving and did not involve large surfaces, electron microscopy could provide snapshots of the suggested splaying of the heads during assembly; not just in mature minifilaments but also in dimers and smaller oligomers. Super-resolution live cell imaging of minifilament assembly, either in cells or in reconstitution assays, could also provide evidence for these intermediate steps. We make the very clear prediction that assembly should start with the antiparallel stagger of 43 nm which already determines the final length of the minifilament. Subsequent growth should then occur at the edges by rolling and zipping. In principle such coordination can be verified in live cell experiments using FRET, but this would be very challenging.

We expect a more specific confirmation of our predictions to come from mutant studies. In the past there have been several studies investigating the ability of NM2 mutants to form minifilaments. Nakasawa and colleagues for example have studied truncated recombinant rod fragments and how well they can assemble into minifilaments (Biochemistry 2005). As the deletion of regions along the rod can be easily incorporated into our model, one could compare predictions for the contact time made by our model to experimentally observed tendencies to form minifilaments. We also think that our work can be used to explain different aspects of minifilament assembly seen in live cell studies. This includes the initial attraction of the positively charged C-terminal tip to the negatively charged area of another rod. Furthermore, as this initial attachment is favorable for almost all staggers, the electrostatic energy is not enough to explain the distinct staggers seen in experiments and the proposed trail-and-error process is a viable mechanism for the rods to assume their optimal configuration. The intermediate sub-optimal configurations, though less likely, should therefore be observable in life-cell experiments. Finally, our model predicts splayed myosin heads and a large degree of staggering disorder even for mature minifilaments as a result of the assembly mechanism.

In response to this question, we have now expanded the corresponding paragraphs in the discussion. On page 13, line 415, we included a comment on mutant studies, and on page 14, lines 439-446, we comment on cellular processes and how to image them. In particular we comment that future advances in electron and super-resolution microscopy might be able to capture the splayed configurations during assembly.

Response to comments by reviewer #2

This theoretical paper considers the configurations of parallel and antiparallel dimers of non-muscle myosin II molecules using the electrostatic and bending energies. Based on the distribution of charge along myosin rods the parallel dimers with

staggers of 14.3, 43.2, and 72 nm present local energy minima. These staggers were previously predicted and observed experimentally. The novelty of this paper is that the authors consider that the length of contact between the two rods is not fully determined by the stagger, but could also change due to bending and splaying of the rods away from each other. Combined electrostatic and bending energy landscape identifies the same favorable staggers, but also suggests that parallel configurations have a strong tendency to splay myosin heads away from the common axis. In contrast, antiparallel configuration with an overlap of 43 nm is very stable and likely serves as nucleus for minifilament nucleation. Using Fokker-Planck equation, the authors compute relative contact times for different configurations and different myosin isoforms suggesting possible order of the assembly of myosin structures in the cell. This approach is novel and elegant, and has important implications for the understanding of the structure and dynamics of myosin II assemblies.

We thank the reviewer for this very favorable evaluation.

I have only one minor comment: given the observations on rich myosin II structure and dynamics that are described in the Introduction, I would appreciate a little more discussion of the relevance of splaying of myosin heads to these variable configurations, e.g. parallel filament stacks.

Our model suggests that the splaying of the myosin heads arises as a natural consequence of the parallel interactions between two NM2 rods. We believe that the splaying is an integral part of the formation of large-scale actomyosin structures. The formation of parallel filament stacks for example is driven by long-range forces "mediated by contractile deformations of the intervening cytoskeletal network" (Dasbiswas et al. Biophysical Journal 2019). These long-range forces require the maximization of the interaction between the NM2 minifilaments and the unordered actin-myosin meshes of non-muscle cells which is effectively enabled by the splaying of the minifilament heads as it allows the minifilament to search its vicinity for actin filaments and to bind several actin filaments at once.

Other processes such as filament splitting or templated nucleation (see Beach et al. Nature Cell Biology 2017) also require single minifilaments to interact with several of the surrounding actin filaments which can be driven by the splaying of the heads.

We completely agree that our model is currently limited by the fact that computationally we can only deal with small systems. In the future, we plan to upscale the model to also deal with large systems, which includes not only more assembled minifilaments, but also realistic models for the complex environments in cells, including different levels of ordering in the actin filaments, the interaction with the intervening cytoskeletal network and super-structures like minifilament stacks. At the same time, we hope that future advances in electron and super-resolution microscopy will make it possible to gather direct evidence for the splayed configurations during assembly, and to correlate them with the staggers.

In response to this comment, we now have extended the corresponding paragraphs in the discussion, in particular on page 14, lines 439-446, where we also addressed a similar comment by reviewer #1.
